# Pediatric myelin oligodendrocyte glycoprotein antibody associated disease—Asymmetric papilledema and elevated ICP are two of the chameleons: A case report

**DOI:** 10.1097/MD.0000000000032986

**Published:** 2023-02-22

**Authors:** Youssef Alqahtani, Mohammed Oshi, Naglaa M. Kamal, Mohammed Aljabri, Salma Abosabie, Waleed Elhaj, Sara A Abosabie

**Affiliations:** a Department of Child Health, College of Medicine, King Khalid University, Abha, Kingdom of Saudi Arabia; b Department of Pediatrics, Neurology Division, Alhada Armed Forces Hospital, Taif, Kingdom of Saudi Arabia; c Pediatrics and Pediatric Hepatology, Kasr Alainy Faculty of Medicine, Cairo University, Cairo, Egypt; d Faculty of Medicine, Julius-Maximilians-Universität Würzburg, Bavaria, Germany; e Faculty of Medicine, Charité Universitätsmedizin Berlin, Berlin, Germany.

**Keywords:** elevated ICP, myelin oligodendrocyte glycoprotein, papilledema

## Abstract

**Background:**

Myelin oligodendrocyte glycoprotein antibody (MOGA) associated diseases are inflammatory immune-mediated demyelinating disorders with relapse potential involving the central nervous system. Multiple unusual clinical manifestations of those disorders were reported, making treatment decisions difficult.

**Case presentation:**

A healthy 12-year-old obese boy presented with headache and bilateral asymmetric papilledema. The patient had a negative medical history. His neurological and general examinations were unremarkable, his initial magnetic resonance imaging showed elevated intracranial pressure (ICP) only. A lumbar puncture revealed increased opening pressure and pleocytosis. The MOGA titer was 1:320. He needed acetazolamide and steroid therapy. After 2 months of medication, weight loss, exercise, the patient symptoms significantly improved, papilledema resolved, and visual function improved.

**Conclusion:**

MOGA-associated disorders have a variety of clinical features, so a high index of suspicion is required for their diagnosis. Papilledema and an elevated ICP are 2 of the chameleons of MOGA-associated disorders. MOGA test may be useful in patients with elevated ICP and inflammatory cerebrospinal fluid profiles. An investigation of the possible association between those disorders and high ICP is warranted.

**Key points**

- Clinicians should have a high suspicion of acquired inflammatory demyelination conditions in cases where IIH is not straightforward, and clinical prevention is atypical.
- This case has highlighted the diverse range of unusual clinical manifestations of MOGA-associated disorders, making the clinical decision to begin treatment difficult.
- Prompt diagnosis is crucial to prevent severe morbidity in MOGA-associated diseases- visual impairment, loss, and further relapses.
- Tests for MOGA and other biomarkers may be useful in patients with elevated.
- ICP and inflammatory CSF profiles.

## 1. Introduction

The acquired inflammatory demyelinating syndromes of the central nervous system (CNS) have recently been expanded to include a unique group of syndromes, associated with myelin oligodendrocyte glycoprotein (MOG) autoantibodies in serum. A variety of syndromes have been proposed with MOG antibodies myelin oligodendrocyte glycoprotein antibody (MOGA). The most common MOGA-associated encephalitis, MOGA-associated optic neuritis,^[1]^ MOGA-associated encephalomyelitis, and others.^[2,3]^

MOG is a myelin glycoprotein expressed on the outermost of the myelin sheath and oligodendrocyte membranes in the CNS, despite the reality that MOG is an extremely minor component of the myelin sheath, and it is been recognized as a putative candidate autoantigen and autoantibody target in demyelination.^[4]^

Multiple studies have shown that MOG-IgG antibody titer, on serial blood analyses, tends to decline in approximately 2-thirds of pediatric patients after an acute incident, especially in those with monophasic acute demyelinating encephalomyelitis (ADEM) like presentation,^[5]^ and in terms of clinical phenotypes of MOGA-associated diseases.

Acute disseminated encephalomyelitis is a well-known phenotype that typically affects children.^[6]^ According to the International Pediatrics Multiple Sclerosis Study Group, a child who experiences their first polyfocal CNS event that is associated with encephalopathy and abnormal magnetic resonance imaging (MRI) of the brain during the acute phase should be diagnosed with ADEM. The spinal cord involvement typically shows confluent lesions. frequently has a favorable prognosis and a monophasic clinical course.^[7]^

MOGA-associated encephalitis can be described as a clinicopathological disorder distinct from the other more common demyelinating phenotypes. Its clinical picture is similar to the diagnostic criteria of possible autoimmune encephalitis.^[8]^ The unilateral hemispheric cortical involvement was described in association with MOGA-associated encephalitis.^[9]^ The second most common phenotype is MOGA-associated optic neuritis with longitudinal extensive optic nerve swelling, usually bilateral, resulting in severe vision loss. Treatment usually results in a visual improvement in most patients.

Asymmetric papilledema is an uncommon finding in idiopathic increased intracranial pressure (ICP). It is defined as an interocular difference of 2 or more Frisén grades. It could be explained by the concept of compartmentation of the perioptic subarachnoid spaces.^[10]^ Transverse myelitis is a recognized clinical phenotype or associated with optic neuritis in a few cases.^[11]^

Several case reports had described MOGA-related disorders in children with elevated ICP who presented with abnormal imaging and/or focal deficits.^[12]^

We describe an extremely rare case of MOGA-related disease who presented with raised ICP, normal brain and spine imaging, and asymmetric papilledema. This could support growing clinical evidence, as well as help in guiding appropriate clinical decision-making.

There can be a misdiagnosis of idiopathic intracranial hypertension with bilateral optic neuritis with disc edema, especially if it is asymmetric, leading to diagnostic delay and serious consequences.^[12]^

We aim to increase the awareness of the unique findings of MOG-IgG-positive optic neuritis, which may initially mimic pseudotumor cerebri, which may lead to delayed diagnosis and treatment.

## 2. Case presentation

A 12-year-old boy, with negative medical history and excellent school performance, presented to the Paediatric Emergency Department at Alhada Armed Forces Hospital, Altaif with 3 weeks of headaches of moderate to severe headache, frontal location, not associated with vomiting, alteration in the level of consciousness, or abnormal movements or seizures, and associated with a reduction in visual acuity and color saturation. No other related symptoms. No history of headache trauma. His birth weight and head circumference were normal. No family history of epilepsy and chronic headache of note.

**Figure 1.**
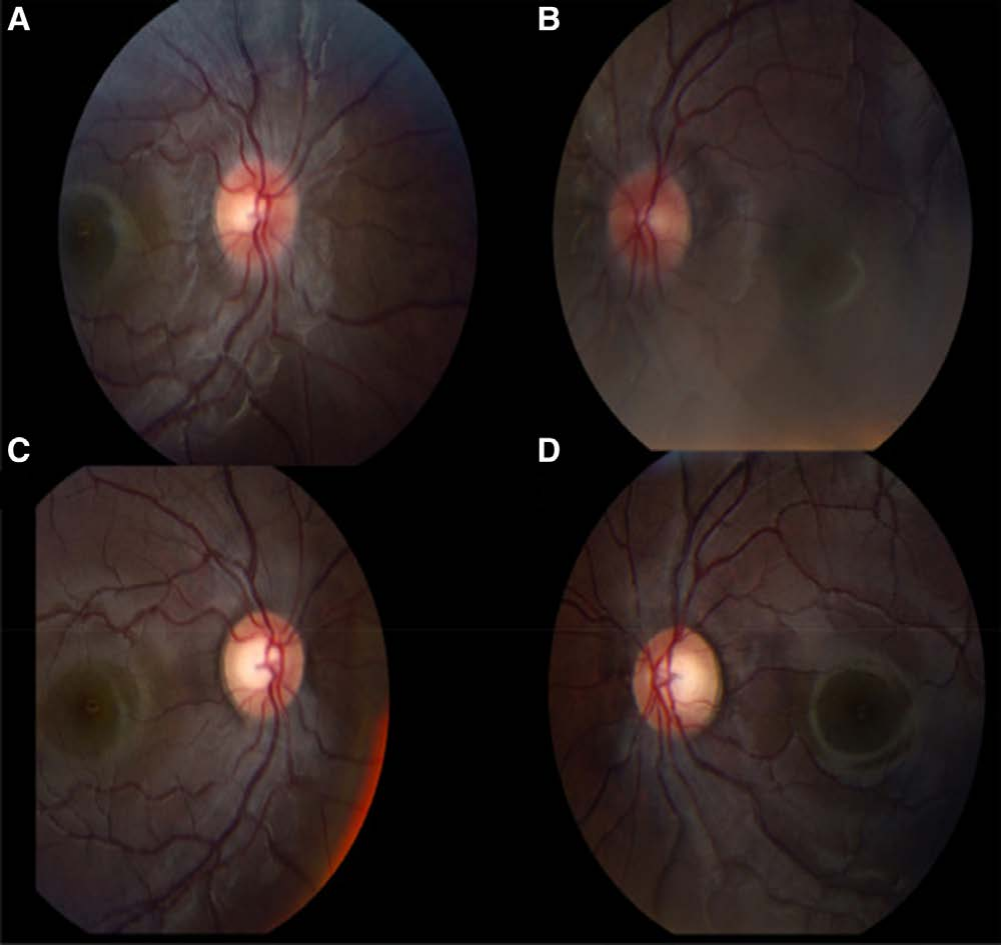
Bilateral papilledema, with some asymmetric right more than left. No clear features of optic neuritis.

Physical examination revealed a conscious child with a weight of 61.6 kg (90th–95th percentile), a height of 145 cm (10th–25th percentile), and a body mass index (BMI) of 28.8 kg/m^2^. He had normal vital signs. His ophthalmologic examination was notable for asymmetric bilateral papilledema (Fig. 1A and B), and visual acuity and visual fields were abnormal. There were no associated painful eye movements, no diplopia, no evidence of abducens nerve palsy, and pupillary light responses were normal. The remaining neurological and systemic reviews were commonplace.

**Figure 2.**
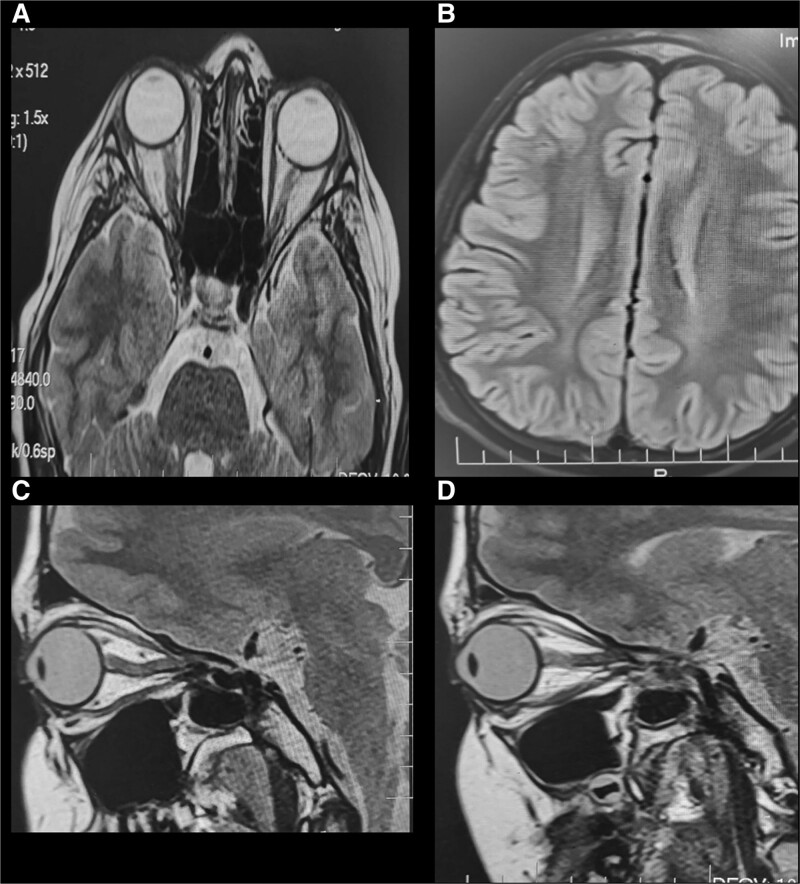
(A) MRI/T2 FLAIR showed evidence of abnormal areas of peripheral enhancement, and increased optic nerve tortuosity, along the course of right optic nerve. (B) Normal brain parenchymal. No lesions (C and D) with some asymmetric right more than left (B). MRI = magnetic resonance imaging. FLAIR = fluid-attenuated inversion recovery.

His initial investigations including complete blood count, renal function test, electrolytes, and liver function test were all unremarkable. Brain computed tomography was normal with no evidence of space-occupied lesions (Fig. 2A and B).

MRI with contrast of the Brain and orbits, showed evidence of elevated ICP, including a flattening of the posterior globes, and prominent both Meckel cave and optic nerves tortuosity (Fig. 2A–D). Spinal cord anatomy was normal with no abnormal signal intensity. No parenchymal abnormalities or areas of enhancement were noted. Magnetic resonance venography showed no venous sinus thrombosis.

Lumbar puncture was performed for the patient with an opening pressure measurement. He had high opening pressure of 35 cm H20. His cerebrospinal fluid (CSF) white blood cell count was 75 (normal range <6) with 89% lymphocytes, 4% neutrophils, 3% monocytes, and 1% eosinophils. CSF chemistry was within normal limits, and cytology was negative.

Based on clinical case profiling and assimilation with CSF pleocytosis, raised ICP and negative herpes simplex virus PCR, and pulse methylprednisolone 30 mg/kg/day was administered for 5 days. After 5 days the patient improved dramatically in terms of the resolution of headache and improved visual function. After that, the patient was started on acetazolamide 10 mg/kg/day and a low dose of steroid. The nonpharmacologic interventions were also started to reduce the weight, including dietary advice and regular physical exercise.

After 1 month, his CSF IgG index was 0.9 (normal range 0.3–0.6), with no oligoclonal bands, and no intrathecal IG synthesis. His autoimmune serology revealed; negative aquaporin 4 antibodies titer (<1:10), and positive MOGA (1:320).

The patient was discharged home on acetazolamide with no steroid taper.

After acetazolamide and steroid for 3 months, his repeated brain and spine MRI showed no abnormalities, he experienced a reduction in headaches and improved quality of life. His most recent clinic visit at 6 months postdischarge revealed no visual symptoms or development of papilledema.

## 3. Discussion

Children with MOGA-associated disorders have significant variations in their clinical picture (1 Even thought that optic neuritis, myelitis, ADEM, and encephalitis are the most common presentation in children, new phenotypes continue to emerge.^[13,14]^

Those patients can be misdiagnosed as idiopathic intracranial hypertension (IIH) due to the associated bilateral optic neuritis with disc edema, especially if it is asymmetric with subsequent diagnostic delay and sericonsequencesence. The CSF pleocytosis and elevated protein rule out this diagnosis.^[15]^

Some patients with headaches and bilateral disc swelling who were initially diagnosed as IIH, were later diagnosed as MOGA-associated disorders after the development of optic neuritis-like symptoms.^[16]^ Their initial MRIs showed optic nerve T2/fluid-attenuated inversion recovery hyperintensities or enhancements, and some showed intraparenchymal T2/fluid-attenuated inversion recovery hyperintensities; this finding is inconsistent with our presentation but different in terms of initial acute visual disturbance and parenchymal lesions, so the disparity between signs and symptoms could delay the diagnosis of serious underlying conditions and the initiation of immune therapy.

Although our patient exhibited high CSF opening pressure similar to Narayan et al,^[17]^ he did not show the typical signs and symptoms of IIH. A diagnosis of optic neuritis was challenging due to the presence of inflammation, severe reduction in visual acuity, visual field defects, and bilateral asymmetric papilledema. An initial MRI revealed no enhancement of the optic nerves, a subsequent MRI revealed patchy areas of the optic nerve on the right side (Fig. 2).

Narayan et al, described 2 cases of MOGA-related disorders, one of which presented with headache, blurred vision, and symmetric papilledema, initially diagnosed as a pseudotumor cerebral syndrome. Mildly elevated cerebrospinal fluid opening pressure and lymphocytosis and the diagnosis of aseptic meningitis was considered. They found longitudinally extensive bilateral optic neuritis and multiple parenchymal lesions on the brain and spinal cord MRI.^[17]^ This is different from our patient in the radiological picture.

The other patient initially presented with unilateral optic neuritis followed 3 weeks later by aseptic meningitis and subsequently ADEM.^[17]^

Several studies suggest that patients with persistent MOG-IgG seropositivity during follow-up are at increased risk of recurrence, requiring prompt diagnosis, long-term follow-up, and prognosis.^[5,18]^

Although it has been taken into consideration in other demyelinating illnesses, the proposed mechanism of elevated ICP in MOGA-associated disorders is currently under investigation.^[19]^ We suggest the same mechanism may be involved in our case, previous studies of MOGA-associated disorders and other demyelinating conditions with elevated CSF pressure, were noted to have very high lymphocytic pleocytosis and significant eosinophil.^[20]^

The CSF WBC in our patient was lower than some other reported cases in the literature (79–157 respectively).^[16,17,19]^

The phenotype in our patient was characterized by asymmetric papilledema that was not described in the aforementioned papers.^[16,17,19]^ He showed earlier in the course of the disease without parenchymal abnormalities, suggesting that there may be another element involved in the genesis of increased ICP. Other CSF dynamic factors might be involved, albeit the cause of these unusual observations is still unknown.

MOGA-associated disorders are treated in the same way as other acquired inflammatory demyelinating conditions, with the most reported first-line therapy being high-dose steroids. Intravenous immunoglobulin, either alone or in combination with steroids is another treatment option. In case of steroid therapy failure, plasma exchange may be advantageous. Our patient responded to an initial high-dose of methylprednisolone with clinical and radiological improvement.

## 4. Conclusion

There are a variety of clinical features associated with MOGA necessitating high clinical suspicion. Papilledema and elevated ICP are 2 of the chameleons of MOGA-related disorders. MOGA testing may be useful in patients with elevated ICP and inflammatory CSF profile. An investigation of the possible relationship between MOGA-related disorders and high ICP is warranted.

## Author contributions

**Conceptualization:** Mohammed Oshi.

**Data curation:** Mohammed Oshi, Naglaa M Kamal, Salma Abosabie, Sara A Abosabie.

**Investigation:** Mohammed Oshi, Mohammed Aljabri, Waleed Elhaj.

**Methodology:** Mohammed Oshi, Mohammed Aljabri, Waleed Elhaj.

**Validation:** Mohammed Oshi, Waleed Elhaj.

**Visualization:** Mohammed Oshi, Mohammed Aljabri.

**Writing – original draft:** Mohammed Oshi, Naglaa M Kamal, Youssef Alqahtani, Salma Abosabie, Sara A Abosabie.

**Writing – review & editing:** Mohammed Oshi, Naglaa M Kamal, Youssef Alqahtani, Salma Abosabie, Sara A Abosabie.
